# Finite State Machine–Guided Retrieval-Augmented Generation Improves Expert-Rated Acceptability of a Peripherally Inserted Central Catheter Self-Management Chatbot: Single-Center Content Validation Study

**DOI:** 10.2196/92374

**Published:** 2026-08-11

**Authors:** Mangyeong Lee, Seung-Beom Cho, Jae-Wook Yu, Junghee Yoon, Jae-Boong Choi, Kyu-Hwan Jung, Juhee Cho

**Affiliations:** 1 Center for Clinical Epidemiology, Samsung Medical Center, Seoul, Republic of Korea; 2 Department of Digital Health, Samsung Advanced Institute for Health Sciences and Technology, Sungkyunkwan University, 115, Irwon-ro, Seoul, Republic of Korea, 82 234101448; 3 School of Mechanical Engineering, Sungkyunkwan University, Suwon, Republic of Korea; 4 Department of Clinical Research Design and Evaluation, Samsung Advanced Institute for Health Sciences and Technology, Sungkyunkwan University, Seoul, Republic of Korea; 5 Department of Medical Device Management and Research, Samsung Advanced Institute for Health Sciences and Technology, Sungkyunkwan University, Seoul, Republic of Korea; 6 Clinical Robotics and Embodied AI Research Center, Smart Healthcare Research Institute, Research Institute for Future Medicine, Samsung Medical Center, Seoul, Republic of Korea; 7 Cancer Education Center, Samsung Medical CenterSeoul, Republic of Korea

**Keywords:** large language models, retrieval-augmented generation, finite state machine, conversational agent, peripherally inserted central catheter

## Abstract

**Background:**

Patients with cancer undergoing long-term or vesicant chemotherapy frequently require peripherally inserted central catheters (PICCs). Due to the nature of ambulatory treatment administration, self-PICC management is essential for the continuation and completion of the planned treatment. Large language models offer potential for continuous patient support, but hallucinations and insufficient adherence to clinical protocols remain concerns. Fine-tuning (FT) and retrieval-augmented generation (RAG) improve factual grounding but cannot enforce the structured decision logic of expert-led PICC consultations, leaving the value of dialogue-control mechanisms unclear.

**Objective:**

This study is an expert-panel evaluation designed to exploratorily verify whether a finite state machine (FSM)–guided RAG architecture yields incremental gains in clinical acceptability when added on top of FT and RAG compared with simpler architectures (fine-tuned model alone; fine-tuned model with RAG).

**Methods:**

We conducted a blinded ablation comparison of three chatbot architectures sharing an identical fine-tuned GPT-4o-mini base: model 1 (fine-tuning only; FT), model 2 (FT + RAG), and model 3 (FT + RAG + FSM). Three nurses specializing in PICC management (≥10 y experience) independently evaluated responses to 43 PICC-related queries. The two-stage evaluation comprised query-level forced-choice preference and global Likert ratings across 8 predefined dimensions. Interrater agreement was quantified with Gwet AC1. Preference rates were compared with Cochran Q and pairwise McNemar tests with Bonferroni correction. Global Likert ratings were summarized descriptively across 8 predefined dimensions.

**Results:**

The FSM-guided model (model 3) was preferred in 34 of 43 scenarios (79.1%). Interrater agreement was moderate (Gwet AC1=0.58, 95% CI 0.42‐0.75; *P*<.001). Differences in model preference were significant (Cochran Q=48.2; *P*<.001). Pairwise McNemar tests showed model 3 was preferred significantly more often than model 1 and model 2 (both *P*<.001), while model 1 versus model 2 did not differ after Bonferroni correction. For the global ratings, descriptive statistics indicated that model 3 received higher ratings than models 1 and 2 across most criteria, although it tended to receive lower ratings for efficiency, possibly reflecting its multiturn structure. In qualitative debriefing, the nurses noted occasional unnecessary conversational turns under FSM guidance.

**Conclusions:**

In this expert-panel content-validation study, the FSM-guided fine-tuned RAG model received higher expert-rated acceptability than the simpler architectures, with FSM-based dialogue control more closely aligning chatbot outputs with expert-led PICC consultation patterns. Patient-facing usability testing and multicenter validation are required before claims regarding patient safety or clinical deployment can be made.

## Introduction

Patients undergoing chemotherapy frequently require peripherally inserted central catheters (PICC) for the administration of vesicant agents or prolonged treatment regimens [1,2]. However, long-term PICC insertion carries a significant risk of complications, including infection and thrombosis. One study reported that approximately half (48.1%) of patients with cancer experienced at least one complication during PICC use [3]. In ambulatory settings, patient competency in catheter management is particularly associated with complications, as evidenced by a decrease in complication rates from 40% to 15.9% following the implementation of PICC management education [4]. While systematic education and counseling for PICC management are crucial, self-care procedures, including disinfection, dressing changes, and heparin flushing, present complex and demanding technical challenges for patients [5]. Consequently, timely assistance is critical when patients encounter concerns or difficulties in catheter management during daily activities [6]. However, patients with PICCs typically face limited access to continuous health care provider communication and subsequently lack adequate support systems to address unexpected situations [7].

Medical chatbot technology has emerged as a promising solution for resolving these unmet needs. Chatbots offer 24-hour availability with immediate response capabilities, enabling prompt assistance when patients experience urgent concerns or discomfort, while providing services independent of physical distance [8,9]. When empathizing with patient-facing concerns and providing personalized advice, chatbots can deliver emotional support, such as anxiety reduction [10,11]. In particular, incorporating large language models (LLMs) can provide superior information quality and user experience compared with conventional instruction delivery [12,13].

Despite these potential benefits, significant concerns and challenges remain regarding the clinical implementation of LLM-based chatbots. The most critical issue is the hallucinations of LLMs. This error manifests as a plausible generation of unfounded content inconsistent with the given questions or contexts, creating risks that general users or patients may misinterpret as factual information [14]. Thus, there is a prevailing perception that rule-based systems remain safer in clinical settings, even within limited scenarios [15]. To address these technological limitations, fine-tuning (FT) and Retrieval-Augmented Generation (RAG) techniques have been considered [16-18]. FT involves additional training with medical domain data to internalize specialized knowledge, whereas RAG produces evidence-based responses through real-time reference to external trustworthy knowledge bases [17,18].

Although FT and RAG can improve factual grounding, they have inherent limitations in structured clinical consultations [18]. Fine-tuned LLMs approximate clinical logic probabilistically and may deviate from the required procedural steps, whereas RAG constrains content but cannot enforce the mandatory sequence or branching rules of guideline-based consultations. In PICC self-management, where symptom triage, safety checks, and decision branching must follow predefined clinical workflows, these limitations result in insufficient procedural validity.

To address this gap, we introduced a finite-state machine (FSM) within a fine-tuned RAG system. An FSM represents consultation as a set of discrete states, such as symptom assessment, severity evaluation, and escalation decisions, with allowed transitions strictly determined by clinical protocols. Constraining the LLM to operate only within verified state transitions preserves the determinism and stability of rule-based systems while retaining the linguistic flexibility of LLMs. Consequently, an FSM-integrated RAG model may offer a more clinically grounded approach for structured consultations, such as PICC self-management. Therefore, this study is a proof-of-concept evaluation designed to exploratorily verify whether FSM-based dialogue control yields incremental gains in clinical acceptability when added on top of FT and RAG for an LLM-based PICC self-management chatbot, using expert content validation as the evaluation method.

## Methods

### Formalization of Consultation Protocol for PICC Management

To ensure comprehensive coverage of PICC-related issues, a knowledge base for a PICC chatbot was designed using educational materials employed at the Samsung Medical Center. These materials comprised general procedures for PICC insertion, potential symptoms or complications during catheter maintenance, and precautionary measures for maintenance, including dressing changes and heparin flushing. Additionally, daily activities prohibited or allowed to keep PICC safe were incorporated into the materials based on questions frequently asked by patients. Most content was similar to other hospitals’ educational materials and was based on patients’ supportive care needs from an empirical study [5,19-23].

To build structured consultation flows, two oncology-specialized nurses were invited to generate response scenarios using a binary decision (yes or no) tree format. For example, when a patient indicated fever symptoms, the guided dialogue was configured to elicit the current body temperature and assess whether it surpassed the threshold of 38°C. Subsequently, if the temperature exceeded the threshold, the manual directed the patients to seek immediate medical attention. All figures and tables within the materials were converted into structured text to enhance the efficiency with which the chatbot processed and retrieved relevant information.

### System Architecture Overview

**Figure 1.**
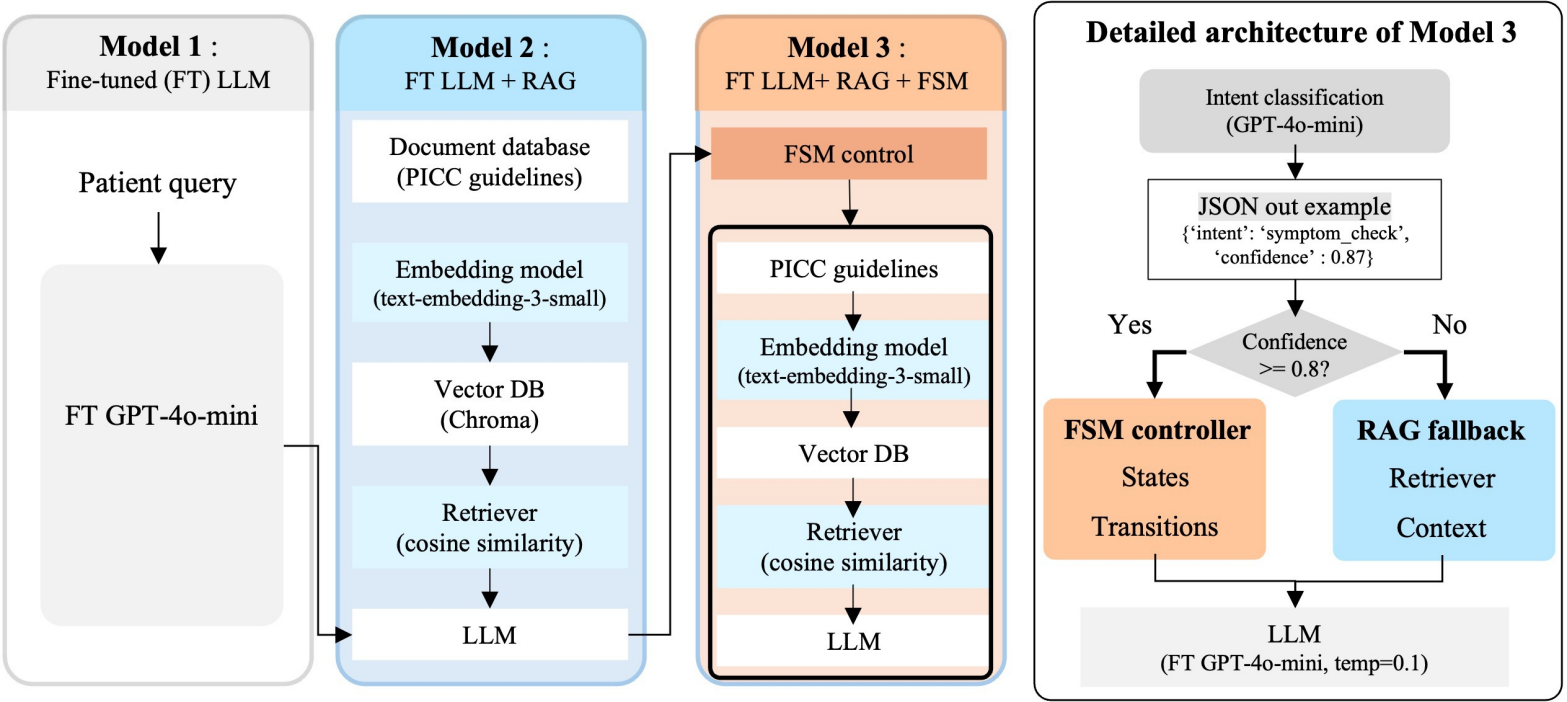
Overall architecture of the peripherally inserted central catheter management chatbot across three experimental settings. Model 1 represents a fine-tuned GPT-4o-mini model trained on guideline-based dialogue data. Model 2 integrates the fine-tuned model with a retrieval-augmented generation system using vectorized guideline documents. Model 3 further extends this architecture by incorporating a finite-state machine for structured dialogue control, enabling contextually accurate and multiturn interactions. DB: database; FSM: finite state machine; FT: fine-tuned; LLM: large language model; PICC: peripherally inserted central catheter; RAG: retrieval-augmented generation.

A multilayered architecture integrating FT, RAG, and a FSM was developed to progressively strengthen protocol adherence in PICC self-management consultations, as illustrated in Figure 1. In model 1, domain-specific responses are generated through FT. In model 2, responses are further grounded in guideline-based documents through RAG. In model 3, an FSM layer is introduced to constrain the dialogue flow according to predefined clinical decision pathways.

User queries are processed by the LLM to generate a JSON-based intent classification and confidence score. When the confidence exceeds a predefined threshold (≥0.8), the query is routed to the corresponding FSM; otherwise, the RAG pipeline is used as a fallback. Through this incremental design, each layer adds a distinct capability—domain knowledge, evidence grounding, and deterministic control—enabling structured and protocol-adherent medical dialogue.

### Incremental Model Construction

To isolate the contribution of each architectural component, the three models were constructed incrementally such that each subsequent model retained all elements of the previous model and added a single functional layer.

#### Model 1: Knowledge Internalization Layer

In model 1, the GPT-4o-mini was fine-tuned to incorporate domain-specific knowledge relevant to PICC patient management. The training dataset was developed by analyzing frequently asked questions derived from PICC-specific educational materials, resulting in 50 dialogue sessions, totaling approximately 300 user-assistant message pairs. Each session consisted of an average of 5‐6 multiturn exchanges to reflect realistic consultation scenarios. FT was conducted using the OpenAI platform, through which the model was optimized to generate responses aligned with the specific domains of PICC care and patient management.

#### Model 2: Addition of a RAG-Based Context Search Layer

Model 2 was constructed by integrating a RAG system with fine-tuned model 1. The RAG mechanism enabled real-time searching of external knowledge bases, which were then incorporated into response generation. This approach provides access to detailed information and guideline updates not included in the FT dataset, thereby enhancing the knowledge coverage and response accuracy [24,25].

The RAG pipeline was implemented using the ”LangChain*”* (LangChain, Inc.) framework with the following configuration. First, the PICC educational materials (originally in PDF format) were extracted using “PyPDF2” (Mathieu Fenniak) and segmented into semantic units through paragraph-level chunking based on double newline delimiters. Each chunk was converted into vector representations using the OpenAI text-embedding-3-small model and stored in a Chroma vector database. Upon receiving a user query, the same embedding model was applied to vectorize the input, after which the top-k most relevant documents (k=3) were retrieved using cosine similarity-based search. The retrieved documents were incorporated into the prompt of the fine-tuned GPT-4o-mini model (OpenAI; temperature =0.1) using a “stuff” chain type via LangChain “RetrievalQA” chain. For multiturn conversations, “ConversationalRetrievalChain” from the LangChain was used, with up to five previous conversation pairs maintained for contextual continuity. A safety mechanism was implemented to provide a “recommend a clinical visit” message when no relevant documents were retrieved.

#### Model 3: Addition of Deterministic Dialogue Control Layer Using FSM

Model 3 was constructed by integrating an FSM-based dialogue flow management system with model 2. The FSM layer encodes PICC-specific clinical workflows using predefined states and transition rules to ensure deterministic and guideline-concordant responses in safety-critical situations [26]. A total of 30 FSMs were constructed based on the consultation protocol prespecified in the Samsung Medical Center, covering nine clinical domains including insertion-site abnormalities, catheter malfunction, symptom assessment, heparin management, and daily activity guidance, as summarized in Table 1. Each FSM encoded a structured consultation flow as a set of discrete states and binary (yes or no) conditional transitions, with 130 states and 72 conditional transitions identified across all FSMs. Conditional transitions represent decision points where the dialogue path diverges based on the patient’s response. FSMs without conditional transitions (eg, FSM-5 and FSM-6) advance automatically through a fixed linear sequence of states, delivering state messages consecutively without branching.

Upon receiving a user query, the system performed intent classification by prompting the fine-tuned GPT-4o-mini model to return a JSON object containing the matched FSM identifier and a confidence score. Queries with confidence ≥0.8 were routed to the corresponding FSM; those below this threshold were directed to the RAG pipeline as a fallback. Within an active FSM session, patient responses at each turn were classified as affirmative or negative through rule-based keyword matching for simple inputs and an LLM-based classification call (temperature =0) for ambiguous responses. If the LLM classification returned “UNCLEAR,” the active FSM session was terminated and the query was directly routed to the RAG fallback pipeline to ensure a timely response. As with all system outputs, the resulting responses were then converted into patient-friendly language through a separate LLM call.

**Table 1.** Overview of the 30 finite state machines (FSM) for peripherally inserted central catheters (PICC) consultation.

FSM^a^ ID	Representative scenario	States	Conditional transitions
Insertion site abnormalities			
FSM-1	Bleeding at insertion site	7	4
FSM-2	Swelling at insertion site	4	2
FSM-3	Pus or discharge	4	2
FSM-4	Skin redness or allergic reaction	4	2
Catheter malfunction			
FSM-5	Catheter torn or punctured	3	0
FSM-6	Catheter dislodgement	5	0
Symptom assessment			
FSM-7	Arm swelling	4	3
FSM-8	Fever after heparin flush	4	3
Care scheduling			
FSM-9 ~ 11	Hospital visit timing, heparin schedule	2–4	0–2
Heparin management			
FSM-12 ~ 15	Storage, dilution, obstruction, home pump	2–3	1–2
Equipment issues			
FSM-16 ~ 18	Fixation device, supplies, hospital transfer	3–5	1–2
General PICC^b^ care			
FSM-19	Duration of catheter use	2	0
Daily activities			
FSM-20 ~ 28	Exercise, bathing, driving, travel, arm use	2–4	0–2
Medical procedures			
FSM-29 ~ 30	Blood draw, blood pressure on PICC arm	2	0
Total			
30 FSMs	—^c^	130	72

a FSM: finite-state machine.

b PICC: peripherally inserted central catheters.

c Not applicable

#### System Prompt Design

A standardized consultation system prompt was applied identically across all three models to control for prompt-related variation and isolate the effect of each architectural component. The prompt instructed the model to assume the role of an oncology-specialized nurse and contained seven directives: (1) listen to and empathize with the patient’s concerns, (2) ask follow-up questions to accurately assess the situation, (3) determine whether the problem is PICC-related, (4) deliver clear warnings about dangerous behaviors, (5) provide guideline-based explanations in patient-friendly language, (6) answer strictly according to medical principles, and (7) assess whether clinical intervention is required. In model 3, two additional prompts were used for FSM-specific functions: an intent classification prompt for routing queries to the appropriate FSM via structured JSON output, and a response naturalization prompt for converting clinical state messages into conversational language. All prompts were written and deployed in Korean. The full text of all prompts, with English translations and API parameters, is provided in Section S1 in Multimedia Appendix 1.

### Expert-Rated Content Validity of LLM-Generated Responses to PICC-Related Queries

**Figure 2.**
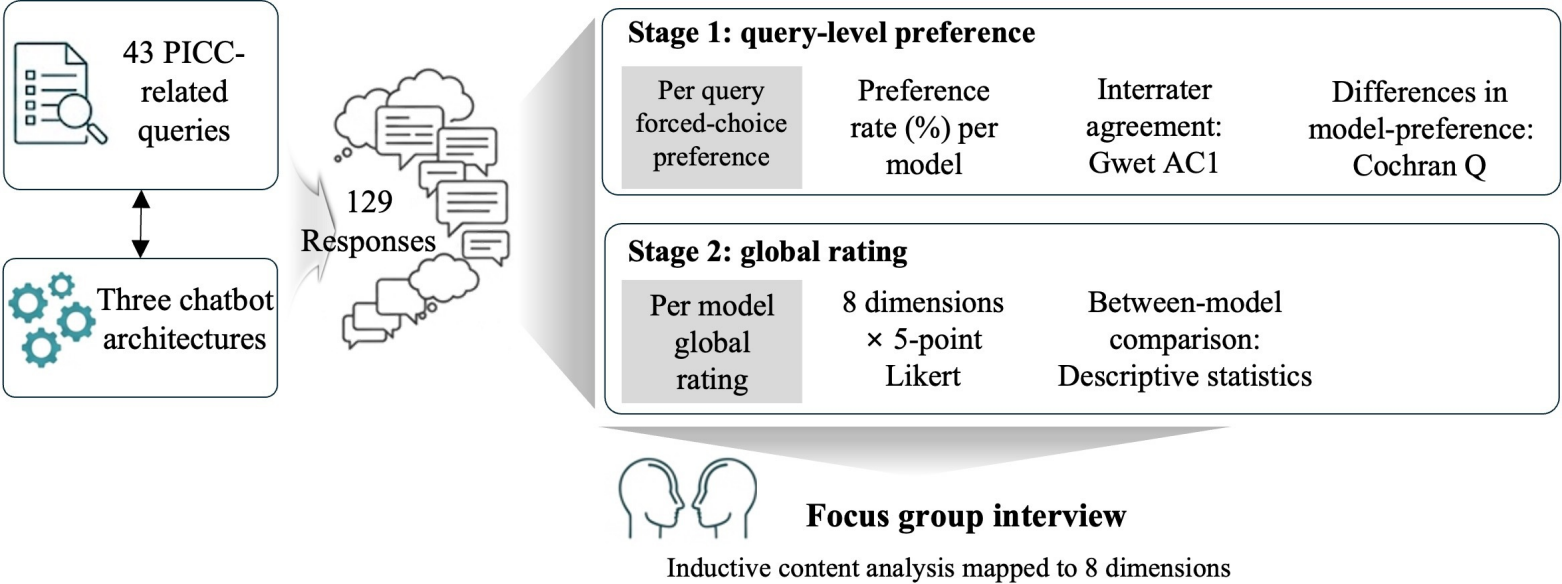
Overview of the expert content validation procedure for LLM-generated responses on PICC self-management. In total, 43 queries were entered into three chatbot models (Models 1, 2, and 3), generating 129 blinded question–response pairs. These were reviewed by three PICC-specialized nurse evaluators. Stage 1 assessed query-level model preference using a forced-choice procedure. Stage 2 elicited global ratings. After reviewing all 43 responses generated by a given model, each expert provided a single global rating on each of the 8 predefined evaluation dimensions using a 5-point Likert scale. A focus group interview was analyzed using inductive content analysis and triangulated with the quantitative findings. LLM: large language model; PICC: peripherally inserted central catheters.

We employed an expert-panel content-validation approach, in which qualified clinical experts judged whether the LLM-generated responses met the standards of expert-led PICC consultation (Figure 2). This approach follows established methodology for clinical content validation, in which panels of three to five highly experienced experts judge whether an instrument or intervention meets domain-relevant clinical standards [27,28]. It is also the modal practice in published LLM-in-health care evaluation, where median panel sizes of 2‐4 evaluators are typical [29].

The evaluation was conducted by a panel of three nurses specializing in infection control and PICC management, recruited via email between August and September 2025, all with at least 10 years of experience in PICC education, counseling, and clinical care for patients with cancer. A query set of 43 PICC-related questions was adopted from a previously published rule-based PICC chatbot whose questions were derived from real patient inquiries [30]. The 43 queries were balanced across six PICC-management-related topics: (1) catheter care (n=10), (2) symptoms (n=4), (3) insertion site abnormalities (n=8), (4) heparin flushing (n=9), (5) daily activities (n=11), and (6) emergency situations (n=1). All identical queries were sequentially entered into the three models (models 1, 2, and 3), generating 129 responses (Section S2 in Multimedia Appendix 1).

The procedure consisted of two sequential stages: a query-level forced-choice preference evaluation, followed by a model-level global rating across eight predefined evaluation dimensions. For each of the 43 queries, each expert independently reviewed the three responses generated by models 1, 2, and 3 and selected the single response judged most appropriate as PICC self-management guidance. To minimize bias, no information regarding the underlying architecture of each model was disclosed to the experts, and all evaluations were conducted in a blinded manner based solely on the response texts. After completing the query-level evaluation for all 43 queries, each expert reviewed the full set of 43 responses generated by a given model and provided a single global rating for that model on each of eight predefined evaluation dimensions: accuracy, clarity, actionability, completeness, efficiency, adaptability, safety, and overall quality. These dimensions were defined based on prior studies of LLM-powered medical chatbots (Textbox 1) [26-31]. Each rating was provided on a 5-point Likert scale (1=highly inappropriate, 5=highly appropriate).

Textbox 1. **Definition of the eight evaluation domains for global ratings.**

1. Accuracy: Is the provided answer medically accurate and compliant with clinical principles in PICC management?
2. Clarity: Is the explanation sufficiently clear and straightforward for patients or caregivers to comprehend immediately?
3. Actionability: Does the response provide specific, executable instructions that patients can practically implement?
4. Completeness: Does the content of the generated response adequately address the patient’s concern (or problem)?
5. Efficiency: Was core information delivered promptly without unnecessary conversational repetition?
6. Adaptability: Does the response consider diverse patient circumstances and provide situation-appropriate answers?
7. Safety: Does the response proactively prevent potential risk factors or patient misunderstandings?
8. Overall Quality: Comprehensive assessment of response quality

### Postevaluation Debriefing: Focus Group Interview

Following the quantitative performance evaluation of the three models, a qualitative focus group interview was conducted as a debriefing session to provide an in-depth interpretation of the evaluation results. The interview was conducted via the Zoom video conferencing platform (Eric Yuan) with the following objectives: (1) to thoroughly understand how each expert interpreted and applied the evaluation criteria, (2) reach a consensus on items where discordance existed among experts, and (3) identify specific areas for improvement, particularly for items that received low scores. The interview lasted for 1 hour and 10 minutes, and the entire session was audio-recorded with the participants’ consent.

### Statistical Analyses

Regarding expert preferences (forced-choice), the frequency of expert-selected models was aggregated to determine the distribution of preferred models per query, and the overall preference rate (%) for each model across all 43 queries was calculated. A winning model for each query was defined as one selected by at least two of the three evaluators. Interrater agreement was quantified with Gwet AC1, which provides more stable estimates than Fleiss’ κ when preferences are imbalanced across categories [31]. Differences in model-preference rates were tested with Cochran Q test, with post hoc pairwise comparisons using McNemar test with Bonferroni correction (corrected α=.0167).

Regarding the global ratings, descriptive statistics were used. All eight domains were presented as mean (± SD). For visual comparison of performance differences among the models, radar charts were constructed based on average scores across the eight evaluation dimensions. All analyses were performed using R (version 4.5.2; Posit PBC). Where inferential statistical tests were conducted, 2-sided tests were used, 95% CIs were reported when appropriate, and *P* values <.05 were considered statistically significant unless adjusted for multiple comparisons using the Bonferroni method.

For the qualitative interview, the recorded material was first transcribed verbatim. Inductive content analysis was subsequently performed on the transcribed data to extract expert rationale and contextual examples that supplemented the quantitative findings. One researcher (ML) organized the coded content, and a second researcher (SB) independently confirmed the coding; discrepancies were resolved by consensus, with adjudication by a senior author (JY) where needed. Through this analysis, we identified the rationale underlying the quality of PICC consultation and the key elements for LLM-based chatbot improvement. The eight evaluation dimensions provided a loose initial framework for organizing codes, but additional themes were allowed to emerge from the narratives.

### Ethical Considerations

This study was reviewed and approved by the Institutional Review Board of Samsung Medical Center (Institutional Review Board number SMC 2025-07-146). All procedures were conducted in accordance with the Declaration of Helsinki. Informed consent was obtained from each evaluator by email prior to participation. Evaluator identities were replaced with anonymized codes (N01, N02, and N03) in all records, transcripts, and reported quotations. However, all evaluator-derived data were temporarily linked back to evaluators throughout the qualitative debriefing interviews. Each evaluator received an honorarium of 100,000 KRW (≈US $70) upon completion of the evaluation.

## Results

### Comparative Evaluation of Information Quality Among Three Models

**Figure 3.**
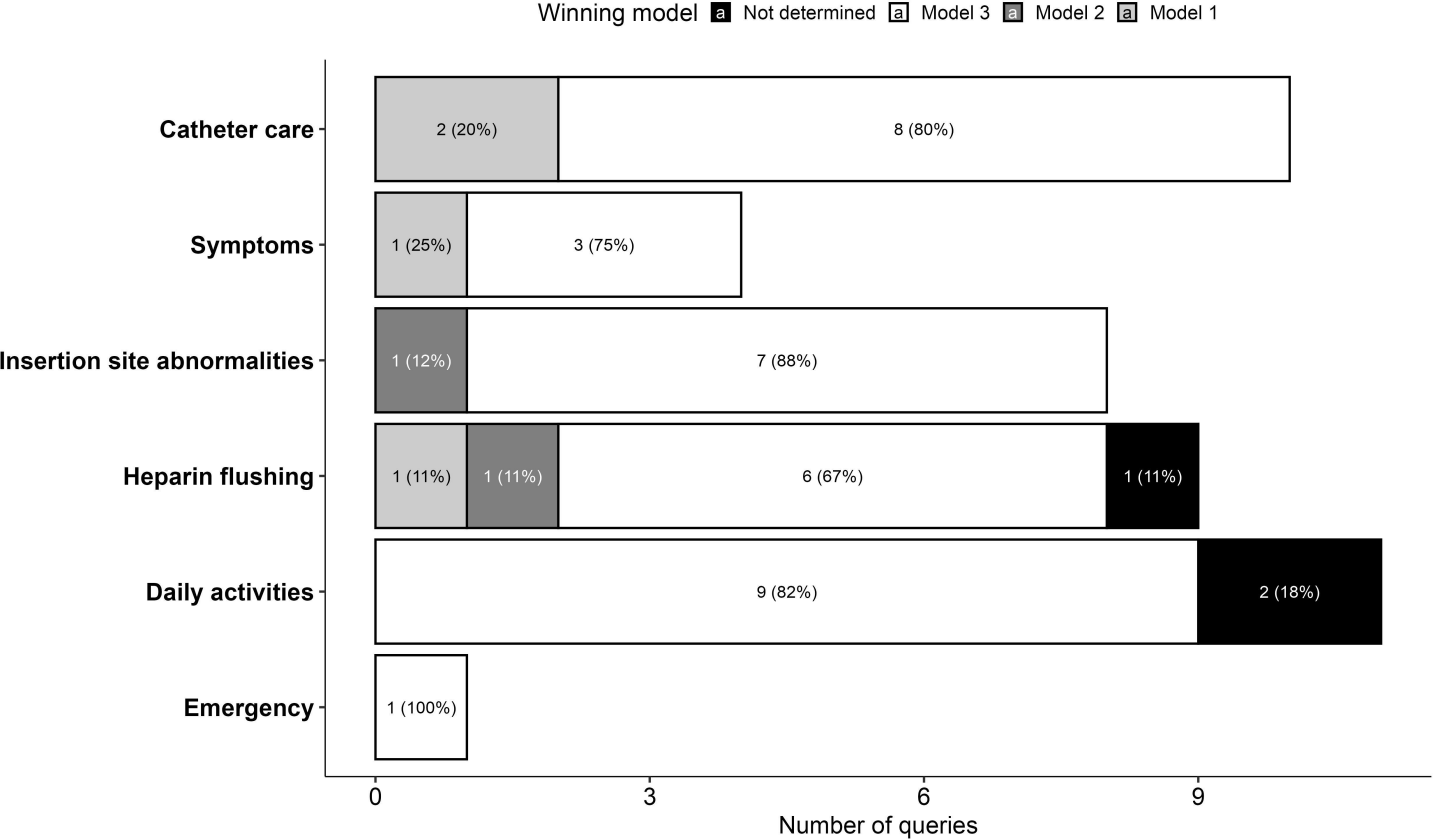
Number of forced-choice expert preferences for each chatbot architecture across 6 categories of peripherally inserted central catheter (PICC)–related management. Catheter care, daily activities, emergency, heparin flushing, insertion site abnormalities, and symptoms. Segments represent responses favoring model 1 (fine-tuned only; FT), model 2 (FT + retrieval-augmented generation [RAG]), and model 3 (FT + RAG + finite state machine [FSM]), with discordance (not determined) indicated separately.

Based on the expert evaluations, model 3, an integrated architecture combining FT, RAG, and FSM, was most frequently selected to provide the most appropriate response across all topics in the PICC query set, followed by model 1 (Figure 3). Model 3 was the most frequently winning model for Catheter care (8/10, 80%), Symptoms (3/4, 75%), Insertion site abnormalities (7/8, 88%), Heparin flushing (6/9, 67%), Daily activities (9/11, 82%), and Emergency (1/1, 100%). However, interrater disagreement was observed in certain categories, particularly for Heparin flushing (1/9, 11%) and Daily activiti*es* (2/11, 18%).

### Query-Level Expert Evaluation Results

**Figure 4.**
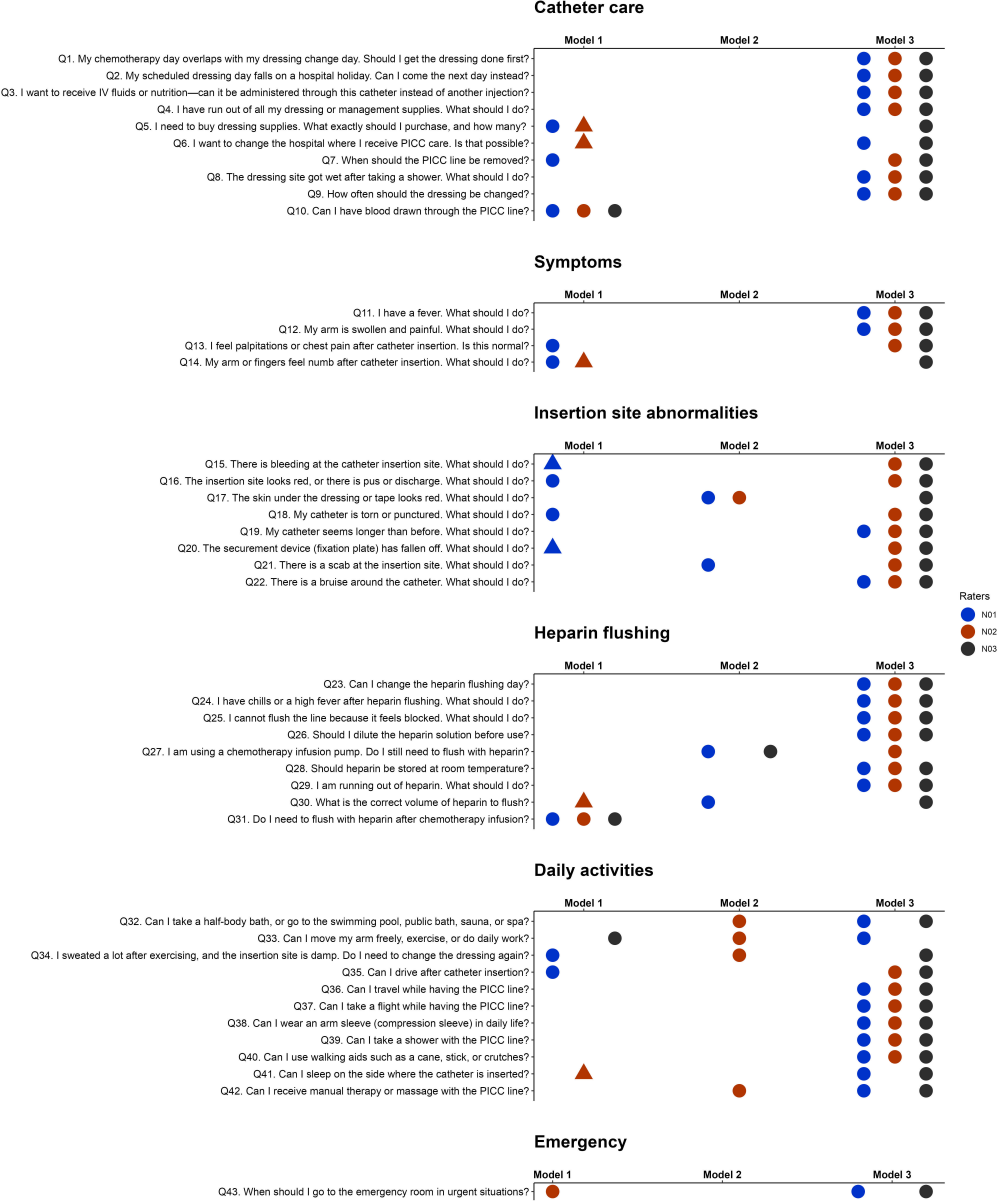
Expert selection of preferred models across queries. The circles represent each evaluator (N01, N02, and N03). Triangles (△) indicate queries for which an evaluator could not determine a preferred model initially and was subsequently requested to make an additional forced choice. IV: intravenous; PICC: peripherally inserted central catheters.

For the 43 query-level forced-choice preference evaluations across the three models, interrater agreement was moderate (Gwet AC1=0.581, 95% CI 0.417‐0.745; *P*<.001). Cochran Q test showed significant differences in evaluator preference frequencies across the three models (Q_2_=48.2; *P*<.001). Post hoc exact McNemar tests with Bonferroni correction indicated that model 3 was preferred significantly more often than model 1 (*P*<.001) and model 2 (*P*<.001), whereas model 1 and model 2 did not differ significantly (*P*=.999; Table S1 in Multimedia Appendix 1). Figure 4 presents a detailed expert preference analysis of all 43 queries. Among these, 23 (53%) achieved a unanimous consensus among the three experts, of which 20 (87%) favored model 3. This pattern indicates that the combined architecture incorporating FSM-based dialogue flow control and RAG-supported knowledge was the most frequently preferred option for the majority of clinically relevant queries.

However, for seven queries, at least one evaluator could not identify a preferred model during the initial evaluation and was subsequently requested to make an additional forced choice. This additional round resolved a determinate winner for six of these queries: Q5 (purchasing dressing supplies), Q6 (changing hospitals for PICC care), Q14 (managing numbness), Q15 (insertion site bleeding), Q20 (securing fixation device), and Q41 (sleeping with catheter). For the remaining query, Q30 (appropriate heparin flushing volume), the three models each received one vote and the outcome remained tied even after the additional forced choice.

Finally, interrater disagreement regarding model preference was observed for three queries: Q30, Q33 (use of the affected arm, physical activity, and daily task limitations), and Q34 (need for dressing replacement due to sweating after exercise). This variability suggests differences in clinical interpretations or context-dependent nuances within certain queries.

### Global Ratings of the Three Models

**Figure 5.**
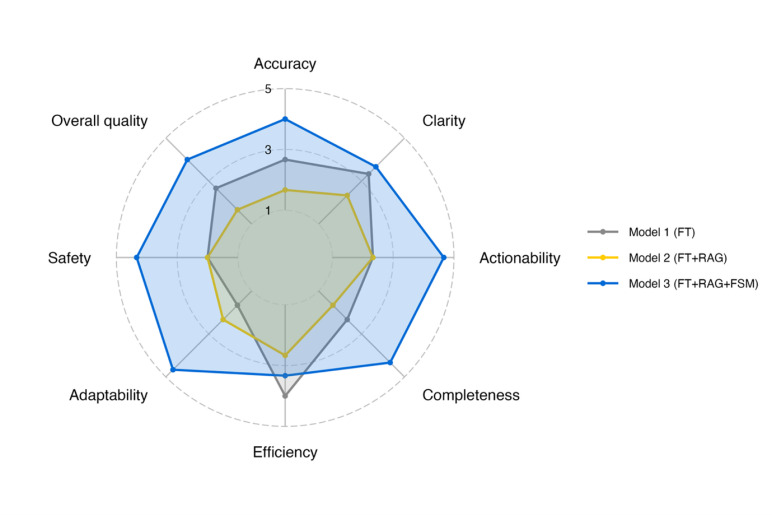
Comparison of global ratings across the 3 chatbot models on 8 evaluation dimensions. Radar chart visualization of expert global ratings (5-point scale) for model 1 (fine-tuned only; FT), model 2 (FT + retrieval-augmented generation [RAG]), and model 3 (FT + RAG + finite state machine [FSM]) across the 8 predefined evaluation dimensions. Each plotted value represents the mean of 3 independent evaluator ratings.

Across the eight evaluation criteria, model 3 received the highest mean ratings for most dimensions in this evaluation (Figure 5). Model 3’s highest ratings were for Actionability (mean 4.67, SD 0.58) and Adaptability (mean 4.67, SD 0.58), while its comparatively lower ratings were for Efficiency (mean 3.33, SD 0.58) and Clarity (mean 3.67, SD 0.58; Table S2 in Multimedia Appendix 1). Compared with model 3, model 1 achieved a higher score for Efficiency (mean 4.00, SD 0.00 vs mean 3.33, SD 0.58). Model 1 showed intermediate ratings overall, whereas model 2 received the lowest ratings, particularly for Accuracy (mean 1.67, SD 0.58), Completeness (mean 1.67, SD 0.58), and Overall Quality (mean 1.67, SD 0.58).

### Expert Debriefing on Key Principles for Improving LLM-Based PICC Consultation Chatbots

Through focus group interviews, experts considered three fundamental components during the quantitative evaluation: (1) in-depth assessment of the patient’s problem and its underlying causes; (2) identification and efficient delivery of information that patients need; and (3) provision of accurate, evidence-based, and tailored responses. These elements were consistently emphasized throughout the discussion as essential criteria for evaluating chatbot performance and determining the appropriateness of consultation quality. The discordance among expert evaluations stems primarily from differing perspectives on the optimal balance between the comprehensiveness and efficiency of information delivery. Nevertheless, based on their rationale for high-quality PICC consultations, evaluators identified several notable strengths in model 3. The experts agreed that the FSM-guided dialogue flow was effective in delivering structured and reliable instructions in clinically careful situations.

The answers (from Model 3) were mostly based on recent evidence and accurately followed the guidelines.

[N01]

When patients use the chatbot for self-care or need to handle things on their own, I feel like model 2 doesn’t really work that well. Model 1’s answers were kind of vague too, so it was probably hard for patients to get clear information. And like she (N01) mentioned, both model 2 and model 1 sometimes didn’t really answer the actual question — they’d kind of go off-topic or talk about something else instead.

[N02]

We’ve used a rule-based chatbot before, but model 3 actually felt more like having a real conversation. There was definitely more of a sense of communicating back and forth.

[N03]

However, model 3 exhibited some limitations that tempered its overall effectiveness. The most prominent concern was the excessive response length with unnecessary ancillary information beyond the core message. In addition, model 3’s assessment-oriented approach was appreciated for its depth, yet criticized for lacking actionable conclusions. Thus, to achieve an optimal balance between assessment depth and response efficiency, they recommended that the system secure more expert cases that include scenario-specific, detailed questioning, and decision-making.

It was like there were so many back-and-forths just to get to that answer. And in the end, it was just about giving the contact info.

[N01]

So with model 3, like I mentioned before, when the chatbot asks about a symptom and the patient says “No,” it just immediately moves on to something like, “Since detailed consultation isn’t possible here, please contact us directly.” There’s no real follow-up or explanation, so the patient might end up thinking “Okay… then why not just call right away?”

[N03]

## Discussion

### Principal Findings

This study showed that a hybrid architecture integrating FT, RAG, and FSM was preferred by expert evaluators in the development of an LLM-based chatbot for PICC patient consultations. In blinded expert evaluations conducted by PICC-specialized nurses, model 3 (FT+RAG+FSM) achieved a preference rate of 79%, showing higher expert-rated acceptability over single-component architecture. In terms of global ratings, model 3 descriptively received higher Likert scores than the other two models on expert-rated actionability and adaptability.

The FSM-based dialogue management system in model 3 was implemented through a two-level hierarchical structure in which the major domains of PICC care, such as symptom assessment, daily management, and emergency conditions, were defined as high-level states. User queries were mapped to appropriate states via intent classification, after which low-level scenario flows generated responses that aligned with clinical protocols. This structure follows the design principles demonstrated in prior work, showing that decomposing complex task-oriented dialogue into subgoals and distributing decision-making across different levels improves response robustness and task completion [32]. The established principles of medical consultation delineate a sequential framework comprising (1) problem identification, (2) comprehensive patient assessment, and (3) the provision of structured and tailored information [33,34]. Considering that PICC consultations must adhere to these established principles, FSM-based response generation may support more clinically acceptable consultation by serving as a “conversational guardrail” that ensures structured, protocol-aligned interactions [35]. Previous research has shown that in medical consultation scenarios, general LLMs used without procedural flow control based on clinical protocols may skip essential symptom assessment steps or generate clinically unverified recommendations [36,37]. Similar patterns were observed in this study, as models without FSM-based control often deviated from the core clinical intent or produced unnecessary information, highlighting the need for structured flow management.

Contrary to our hypotheses, model 2 (fine-tuned model + RAG) received lower descriptive Likert ratings than model 1 (fine-tuned model alone) on overall quality and most evaluation dimensions. This may be because the contextual volume of the educational materials used in this study was insufficient. In general, the RAG was invented to address the hallucinations of LLMs; however, its performance can be affected by the quality of documents retrieved by LLMs [38]. Notably, when documents contain insufficient contextual information to disambiguate vague or imprecise user queries, contradictory or distracting material is likely to be incorporated during the retrieval process, thereby diminishing the accuracy of LLM-generated responses [39]. Previous research has demonstrated that the RAG system performance declined below that of the baseline model operating without a retrieved context when a single irrelevant document was incorporated into the retrieval set [40]. Although numerous hospitals provide patients and caregivers with PICC education materials, the instructions are typically brief to ensure ease of understanding [19-22]. Hence, it may have been relatively difficult for the RAG, which uses simplified documents, to provide quality and sufficient information based on the retrieved context (poor chunking quality). In addition, our retrieval was configured with top-k=3 without a similarity threshold or no-retrieval fallback. Hence, under sparse and brief source documents, this likely surfaced topically adjacent but non-gold chunks that were nonetheless injected into the prompt (poor retrieval relevance). The combination of these two mechanisms—poor chunking quality and poor retrieval relevance—may explain why model 2 fell below the model 1 baseline rather than defaulting to it. From this perspective, our findings suggest that integrating FSM frameworks into LLMs may mitigate existing technological limitations by establishing more deterministic conversational pathways tailored to individual patient inquiries, even when the underlying document repository is insufficient.

Despite its strengths, model 3 exhibited difficulties with several query types. These queries tend to involve context-dependent considerations tightly linked to real-world daily routines or individualized patient circumstances, domains in which flexible reasoning beyond the constraints of FSM rules is often required. Moreover, these cases frequently lack a single correct answer within the clinical guidelines, requiring experiential judgment from experts. Variability in expert interpretation, shaped by differing clinical priorities, such as emphasizing reassurance versus patient convenience, also contributed to disagreement in those cases. A related concern raised during expert evaluation was conversational efficiency. This is because the current FSM design elicits one piece of information per turn; clinically straightforward scenarios sometimes required more dialogue steps than necessary. A potential solution is multislot extraction, in which the LLM is prompted to identify multiple relevant entities (eg, symptom type and severity) within a single conversational turn, thereby reducing the number of required exchanges while preserving protocol adherence. This optimization, combined with adaptive turn-skipping logic that bypasses redundant assessment steps when sufficient information is already available, represents a promising direction for improving the practical usability of FSM-guided dialogue systems.

From a technical standpoint, the development of FSM-based states and transition rules requires substantial expertise and iterative refinement. Mapping diverse query types to predefined states and specifying appropriate transition conditions necessitate close collaboration between clinical experts and system developers, which may limit scalability and increase the system maintenance burden. To address these challenges, future systems may benefit from three key strategies: (1) proactive collection of expert-derived real-world cases, (2) transformation of these cases into formalized ontologies, and (3) the development of interpretable reasoning modules grounded in such ontologies.

Ontologies can structure hierarchical and semantic relationships between clinical concepts, partially automating intent classification and state mapping, while enhancing system extensibility [41-43]. When integrated with standardized medical ontologies, the reliance on manually defined rules can be reduced while preserving clinical accuracy. Furthermore, ontology-based approaches offer reusable knowledge structures that support efficient scalability beyond PICC management to other clinical domains.

### Limitations

This study had several limitations. First, the evaluation was based on human expert assessments, and no benchmark dataset currently exists for validating PICC-related chatbot performance. The evaluation criteria were adapted from prior studies but were not derived from a formally validated instrument, limiting the ability to derive fully objective quantitative measures. Accordingly, future research should prioritize the development of dedicated evaluation metrics for LLM-mediated clinical consultations or, alternatively, adapt established frameworks, such as the Calgary–Cambridge Guide, to assess adherence to the principles of effective patient–physician communication [33,34].

Second, although the assessment was conducted blinded, true blinding may have been severely compromised by the structurally distinct outputs of the FSM-guided model, which generated noticeably longer responses with multiturn back-and-forth interactions. While the evaluators received no prior briefing on the underlying technical mechanisms, the structural distinctness of the FSM-guided outputs may have inadvertently signaled the architecture in some cases. The expert preference for the FSM-guided model should therefore be interpreted with this caveat.

Third, model performance was constrained by the scope of the preconstructed educational materials used for training. Although these materials sufficiently cover core PICC management tasks, the system may be limited in responding to diverse real-world scenarios encountered in everyday life. Therefore, additional strategies are required to support flexible reasoning in unexpected and context-rich situations. In addition, the knowledge base and FSM transition rules were derived from educational materials and clinical workflows of a single tertiary cancer center. Institution-specific elements such as dressing intervals, role permissions for heparin flushing, and local escalation pathways may not generalize to settings with different staffing models or referral practices. The 43 evaluation queries, although derived from real patient inquiries, were collected at the same single cancer center, which may also limit their generalizability. Multicenter validation would be required before the proposed architecture can be considered clinically generalizable.

Fourth, the RAG chunking strategy used in this study relied on double-newline (paragraph-level) delimiters matched to our institutional materials, which were preformatted with consistent paragraph breaks. Given that PDF text extraction can introduce irregular or broken newline characters, this approach is sensitive to document formatting and may have reduced chunking quality. A more sophisticated, layout-aware parsing strategy might therefore have yielded different baseline results for model 2, and the chunking approach would need to be adapted before applying this architecture to external document sources.

Fifth, the evaluation relied solely on health care professionals and may not have fully reflected patient-perceived usefulness or usability. Establishing content validity by qualified clinical experts was a deliberate precondition of this study. It is crucial to ensure the safety of content before it is delivered to patients and to enable subsequent patient-experience data to be interpreted against a clinically validated baseline. Building on this expert-validated baseline, future work should extend the evaluation to include patient-facing usability assessment together with operational performance metrics such as system latency, token costs, and fallback frequency.

### Conclusions

In conclusion, an FSM-guided RAG architecture (model 3) was preferred by oncology nurses in 79% of PICC self-management scenarios over a fine-tuned–only model (model 1) and a fine-tuned+RAG model (model 2). Model 3 received descriptively higher expert-rated acceptability scores on actionability, adaptability, and safety, while showing lower ratings on efficiency, possibly reflecting its multiturn structure. Model 2 received descriptively lower Likert ratings than model 1 on most dimensions, although the two single-component architectures did not differ significantly in 43-query model-preference rate after Bonferroni correction. These results indicate that retrieval augmentation alone, without an additional structural constraint, did not yield a robust improvement in expert-rated acceptability in this single-center evaluation. These findings support the incremental value of FSM-based dialogue control as a complement to FT and a RAG system. By constraining the conversation flow to state transitions based on clinical evidence, the chatbot’s output becomes more closely aligned with established expert PICC consultation patterns. While this study did not assess patient-facing usability, our findings suggest the potential to provide more clinically plausible virtual consultations to patients.

## Supplementary material

10.2196/92374 Multimedia Appendix 1 .Supplemental materials.

## Abbreviations

FSM: finite-state machine
FT: fine-tuning
LLM: large language model
PICC: peripherally inserted central catheter
RAG: retrieval-augmented generation
