# Supplementary material for: Finite State Machine–Guided Retrieval-Augmented Generation Improves Expert-Rated Acceptability of a Peripherally Inserted Central Catheter Self-Management Chatbot: Single-Center Content Validation Study
Source: J Med Internet Res. 2026 Aug 11;28:e92374. doi: 10.2196/92374 (PMC13460064; doi:10.2196/92374)
Supplement: Supplementary file 1 [file jmir-v28-e92374-s001.docx]

**Supplementary Information**

**Section S1. System Prompt Used Across All Three Chatbot Models**

**Section S2. All queries used in this study and responses generated by the three models.**

**Supplementary Table S1.** **Pairwise exact McNemar tests for forced-choice evaluator preferences across the three models.**

**Supplementary Table S2. Quantitative performance comparison across eight evaluation criteria.**

**Section S1. System Prompt Used Across All Three Chatbot Models**

The following system prompt was applied identically to Models 1, 2, and 3.

**Prompt 1. Consultation System Prompt (Applied to Models 1, 2, and 3)**

This prompt defined the chatbot's clinical role and communication guidelines. It was applied identically across all three models to ensure that any observed performance differences were attributable to architectural components rather than prompt variation.

You are an oncology-specialized nurse. Your role is to provide information and counseling to cancer patients with peripherally inserted central catheters (PICC) undergoing chemotherapy regarding daily catheter management. Please conduct consultations as follows:

1. Listen to the patient's questions or situation and respond empathetically.
2. When necessary, ask follow-up questions to more accurately assess the patient's condition or situation.
3. Carefully determine whether the patient's problem or question is related to PICC management.
4. Deliver clear warnings about potentially dangerous behaviors.
5. Base your responses on the retrieved context, but explain them in a way that is easy for patients to understand.
6. Answer strictly according to medical principles.
7. Consider whether clinical intervention by medical staff is required when providing your response.

Answer the following question based on the retrieved information.

Retrieved information: {context}

Question: {question}

**Prompt 2. Intent Classification Prompt (Model 3 Only)**

This prompt was used in the IntentMapper module to classify user queries and route them to the appropriate FSM. The LLM was instructed to return a structured JSON object containing the matched FSM identifier, confidence score, urgency level, and reasoning. The {fsm_list} placeholder was dynamically populated with the complete catalog of 30 FSM IDs, names, and descriptions at runtime.

You are a medical AI that identifies the intent of PICC (peripherally inserted central catheter) patients and maps them to the appropriate FSM (Finite State Machine).

FSM catalog:

{fsm_list}

Analysis guidelines:

1. First, distinguish whether the patient's statement concerns a symptom, a care inquiry, or a usage inquiry.
2. If symptom-based, prioritize emergency FSMs; if care- or usage-related, map to the corresponding FSM.
3. Assign a confidence score precisely between 0.0 and 1.0.
4. If the query is vague or ambiguous between FSMs, assign confidence below 0.8.
5. The confidence score should reflect the actual validity of the FSM mapping; do not overestimate, and provide specific reasoning.
6. In the reasoning field, explain why the FSM was selected in as specific terms as possible (within 100 characters).

Patient input:

"{user_input}"

Response format:

json

{

"main_purpose": "Most appropriate FSM name",

"fsm_id": "FSM-XX",

"sub_keywords": ["keyword1", "keyword2"],

"urgency": "high/normal/low",

"confidence": 0.0-1.0,

"reasoning": "Detailed explanation of mapping rationale",

"extracted_phrases": ["Key phrases from the patient's input"],

"should_use_fsm": true/false

}

Respond only in valid JSON format. Do not include any explanations or comments.

**Prompt 3. Y/N Response Classification Prompt (Model 3 Only — FSM State Transitions)**

This prompt was used within active FSM sessions to classify patient responses as affirmative (Y) or negative (N) at each state transition. It was invoked only when rule-based keyword matching failed to resolve the patient's intent. The {current_question} and {patient_response} placeholders were dynamically populated at each conversational turn.

System prompt: You are a medical AI that accurately classifies patient responses as Y/N.

User prompt: Analyze whether the patient's response is affirmative (Y) or negative (N).

Current question: "{current_question}" Patient response: "{patient_response}"

Analyze according to the following criteria:

1. Explicit single-character responses such as "Y", "N", or "yes", "no" should be interpreted directly.
2. Expressions indicating the occurrence of a problem (e.g., "it fell off") should be classified as Y.
3. Expressions indicating the absence of a problem (e.g., "it didn't fall off") should be classified as N.
4. If the question asks about a specific area (e.g., "the edge") but the patient mentions a different area (e.g., "the center"), classify as N.
   - Example: If the question is "Did the edge come off?" and the patient says "No, the center," classify as N.
   - Example: If the question is "Did the edge come off?" and the patient says "Not the center," classify as Y.
   - Example: If the question is "Did the edge come off?" and the patient says "Only the center came off," classify as N.
5. If the response contains both affirmative and negative elements, prioritize the more emphasized meaning.
6. If the intent is unclear, consider the context of the current question to make a judgment.
7. Special cases to note:
   - "Not the center" is a clear negative (N) in response to an center-related question.
   - "Not the edge" or "it's not the edge" is a clear negative (N) in response to an edge-related question.

Respond with only Y or N. Do not include any other text.

Only respond with "UNCLEAR" if analysis is completely impossible.

**Prompt 4. Response Naturalization Prompt (Model 3 Only)**

This prompt was used to convert FSM state messages (which were written in clinical shorthand) into patient-friendly conversational language. It was applied to every FSM output before delivery to the patient.

System prompt: You are an expert in converting medical dialogue into natural conversational language.

User prompt: Convert the given medical question/state message into a more natural and empathetic conversational tone.

Original message: "{message}"

Follow these guidelines for conversion:

1. Use a tone that is not overly mechanical, as if casually speaking to the patient without creating pressure.
2. Replace medical terminology with easy and familiar expressions whenever possible.
3. For questions, distill them to simple, focused questions containing only the essential point. Do not include unnecessary additional questions.
4. Do not use expressions like "Please answer with yes or no." Instead, rephrase as natural interrogative sentences.
5. For informational messages, convert them into kind and gentle explanations.

Output only the converted sentence. Do not include any other explanations.

**Section S2. All queries used in this study and responses generated by the three models.**

| Q# | Queries | Model 1 (FT only) | Model 2 (FT + RAG) | Model 3 (FT + RAG + FSM) |
| --- | --- | --- | --- | --- |
| **Q1** | My chemotherapy day overlaps with my dressing change day. Should I get the dressing done first? | If your chemotherapy day and dressing change day overlap, you should do the dressing change first. You can receive chemotherapy within 24 hours after the dressing change. | Your dressing change day and chemotherapy day overlap. If you skip the dressing change, the catheter may become occluded. You should proceed with the dressing change. | Your dressing change day and chemotherapy day overlap. You can just come in for treatment without doing a separate dressing change. You don't need to do dressing separately. |
| **Q2** | My scheduled dressing day falls on a hospital holiday. Can I come the next day instead? | If you cannot do the dressing change on the scheduled day due to a hospital holiday, it is best to have it done as soon as possible. It is fine to have it done the next day. | If your scheduled dressing day falls on a hospital holiday, it is recommended to have the dressing done in advance before the holiday. | Is the hospital open the day before your scheduled care day? No. Catheter dressing and heparin flushing should be done within 7 days. If the day before is also a holiday, it is better to have your care done one or two days earlier. |
| **Q3** | I want to receive IV fluids or nutrition—can it be administered through this catheter instead of another injection | Yes, IV fluids or nutrients can be administered through the PICC line. There is no need for a separate injection. | Yes, IV fluids or nutrients can be administered through this catheter. There is no need for a separate injection. | Yes, it is possible. You can receive IV fluids or nutrients through this catheter. However, you need to confirm that the hospital knows how to handle the catheter. After the infusion, heparin solution should be flushed to prevent catheter occlusion. |
| **Q4** | I have run out of all my dressing/management supplies. What should I do? | If your dressing/care supplies are depleted, you should visit a nearby pharmacy or hospital to purchase the necessary items. It is best to prepare in advance so that dressing changes are not delayed. | If your dressing and care supplies have been depleted, you should contact the hospital to purchase additional supplies. | Have you tried visiting a nearby pharmacy or medical supply store? No, I haven't. You could try visiting a nearby pharmacy or medical supply store to purchase them. There aren't any near me. If there are no nearby pharmacies or medical supply stores, you can visit or call our hospital's medical supply store for delivery. The phone number is 02-3410-0500. |
| **Q5** | I need to buy dressing supplies. What exactly should I purchase, and how many? | For dressing supplies, you will need 10mL syringes, heparin solution, alcohol swabs, and transparent film. Purchasing 2-3 of each item would be convenient for dressing management. | For catheter dressing, you will need alcohol swabs, transparent film, and heparin solution (if needed). It is recommended to purchase at least 5 of each item. | Have you tried visiting a nearby pharmacy or medical supply store? I haven't visited a pharmacy or medical supply store, but I'm curious about how many of each to buy. You could try visiting a nearby pharmacy or medical supply store to purchase them. |
| **Q6** | I want to change the hospital where I receive PICC care. Is that possible? | If you want to change your care hospital, you should contact the current hospital to learn about dressing methods and supply information in advance. Then visit the new hospital for dressing changes. | You want to change your care hospital. Please let us know the hospital name and contact information you wish to change to, and we will guide you through the necessary procedures. | The dressing methods may differ slightly. Are you thinking about changing hospitals? Yes, it's too far. If you want to change hospitals, you need to contact the Partners Center. Would you like the contact information? Yes. Please contact the Partners Center at 02-3410-0500. Consultation is available on weekdays from 9 AM to 5 PM. |
| **Q7** | When should the PICC line be removed? | The PICC line is removed when chemotherapy is completed and it is no longer needed. Please discuss the removal timing with your attending physician. | Catheter removal is determined by the attending physician. The catheter will be removed when chemotherapy is completed. | The central venous catheter will be removed at an outpatient visit after chemotherapy is completed. Since this catheter was inserted for chemotherapy administration, it is no longer needed after treatment ends. Are you currently undergoing chemotherapy? Yes, I am. After chemotherapy ends, the medical team will check your condition and remove the central venous catheter. |
| **Q8** | The dressing site got wet after taking a shower. What should I do? | If the dressing site got wet after showering, you must redo the dressing. Clean the area with alcohol swabs and reapply the transparent film according to the dressing procedure. | If the dressing site got wet after showering, you must redo the dressing. Can you safely proceed with the dressing in a dry location? | If the dressing site got wet after showering, you must redo the dressing. First, dry the wet area with tissue and then proceed with disinfection. |
| **Q9** | How often should the dressing be changed? | PICC catheter dressing should be done every 7 days. Plan your dressing schedule in advance and follow it consistently. | Dressing should be done regularly every 7 days. Also, the first dressing should be done within 24 hours after catheter insertion. | Dressing should be done regularly every 7 days. Also, always keep the dressing site dry and check that the dressing material is well adhered after each change. Do you have any more questions about the dressing schedule or method? |
| **Q10** | Can I have blood drawn through the PICC line? | Yes, blood can be drawn through the PICC line. After blood draw, flush with heparin solution to prevent catheter occlusion. | Yes, blood can be drawn through the catheter. Don't forget to do the dressing after blood draw. | Blood can be drawn through the catheter. You can have blood drawn from the arm with the central venous catheter. |
| **Q11** | I have a fever. What should I do? | If you have a fever, first check the catheter management status to determine whether the fever may be due to catheter-related infection. Verify that dressing has been done properly and consult with medical staff if necessary. | If you have a fever, there may be a risk of infection. If it is 38°C or higher, contact 1599-3114 for medical advice. | Is your fever 38°C or higher? [If yes:] Please visit the hospital within 24 hours. Chemotherapy can lower your white blood cell count, increasing infection risk. Please visit the emergency room to identify the cause of the fever and receive appropriate treatment. [If no:] Can you recheck your temperature in one hour? If it remains above 38°C, you may need to visit the emergency room. |
| **Q12** | My arm is swollen and painful. What should I do? | If your arm is swollen and painful, there may be a catheter-related problem. Check the catheter management status and consult with medical staff for appropriate measures. | If your arm is swollen and painful, there is a possibility of blood clot formation. In this case, contact 1599-3114 for medical advice. | Is only the arm where the catheter is inserted swollen and painful? [If yes:] Is the swollen arm red or warm? [If no redness:] For more detailed consultation, please contact the hospital at 1599-3114. [If both arms:] If both arms are swollen, it is likely not caused by the catheter insertion. There may be another cause that requires further examination. |
| **Q13** | I feel palpitations or chest pain after catheter insertion. Is this normal? | If you experience chest palpitations or pain after catheter insertion, this may be a catheter-related issue. Consult with medical staff immediately to identify the cause and take appropriate measures. | If you experience chest palpitations or pain, there may be a cardiac issue. Contact 1599-3114 for medical advice. | These symptoms can sometimes occur when the inserted catheter is too deep. Visiting the outpatient clinic for catheter position adjustment may relieve the discomfort. Please contact our hospital at 1599-3114 for detailed consultation. |
| **Q14** | My arm or fingers feel numb after catheter insertion. What should I do? | If your arm or fingers feel numb after catheter insertion, this may be due to catheter positioning or occlusion. Check the catheter management status and consult with medical staff. | If your arm or fingers feel numb, there is a possibility of blood clot formation. Contact 1599-3114 for medical advice. | Is only the arm where the catheter is inserted swollen and painful? Yes. Is the swollen arm red or warm? No. For more detailed consultation, please contact the hospital at 1599-3114. |
| **Q15** | There is bleeding at the catheter insertion site. What should I do? | If there is bleeding at the insertion site, take appropriate measures depending on the severity. For minor bleeding, disinfect and re-cover with transparent film. For continued or severe bleeding, consult medical staff. | If there is bleeding at the insertion site, you need to re-disinfect. If the bleeding is not severe, please proceed with disinfection. For severe bleeding, contact medical staff. | Have you done the dressing? [If no:] Apply Medix pad to the disinfected area. [If yes:] Is the bleeding still continuing after dressing? [If no:] Do the dressing again in two days and apply transparent film afterward. |
| **Q16** | The insertion site looks red, or there is pus/discharge. What should I do? | If the insertion site is red or has pus or discharge, this may be a sign of infection. Check the dressing status and consult with medical staff for appropriate measures. | If the insertion site is red or has pus or discharge, this may be a sign of infection. Contact 1599-3114 for medical advice. | Have you been experiencing any pain recently? [If no pain:] Please visit a nearby hospital for re-disinfection with Medix. |
| **Q17** | The skin under the dressing or tape looks red. What should I do? | If the skin around the dressing material or bandage area has become red, this may be an allergic reaction or sign of infection. Check the dressing status and consult with medical staff if necessary. | If the skin around the dressing material or bandage area has become red, this may be an allergic reaction. In this case, try re-disinfecting with a different dressing material. | Has the disinfected area turned red? [If no:] For more detailed consultation, please contact the hospital at 1599-3114. |
| **Q18** | My catheter is torn or punctured. What should I do? | If the catheter is torn or has a hole, you must visit a nearby hospital or our hospital immediately to have the catheter removed. Consult with medical staff about catheter replacement or removal. | If the catheter is torn or has a hole, chemotherapy agents may leak. Contact 1599-3114 for medical advice. | Can you move the clamp above the torn or punctured area and close it? [If no:] Please visit a nearby hospital or our hospital as soon as possible. |
| **Q19** | My catheter seems longer than before. What should I do? | If the catheter appears to have gotten longer or extended, there is a risk of catheter dislodgement. Consult with medical staff immediately for appropriate measures. | If the catheter appears to have gotten longer or extended, there is a risk of dislodgement. Contact 1599-3114 for medical advice. | A longer catheter may indicate dislodgement or extension. If extended, there is a risk of the catheter falling out, so it is important to contact medical staff immediately. If dislodged, prompt action is needed—visit a nearby hospital for medical assistance. |
| **Q20** | The securement device (fixation plate) has fallen off. What should I do? | If the fixation device has come off, there is a risk of catheter dislodgement. Consult with medical staff immediately for re-fixation. | If the fixation device has come off, there is a risk of catheter dislodgement. Contact 1599-3114 for medical advice. | Did only the edge of the fixation device come off? [If yes:] Please secure the detached area with adhesive tape to prevent further detachment. [If the whole thing came off:] Did the entire fixation device at the insertion site come off? [If yes:] Since other hospitals may use different fixation methods, it is best to visit our hospital for replacement. |
| **Q21** | There is a scab at the insertion site. What should I do? | If there is a scab at the insertion site, protect the area by applying transparent film after disinfection. For severe scabbing, consult with medical staff. | If there is a scab at the insertion site, disinfection is needed. After disinfection, be careful to prevent the scab from forming again. Can you safely proceed with the disinfection? | If a scab has formed at the insertion site, first check whether there is any further bleeding. If no more bleeding, gently remove the scab with a disinfection swab during the next dressing change. If bleeding continues or does not stop, please consult with medical staff. |
| **Q22** | There is a bruise around the catheter. What should I do? | If there is bruising around the insertion site, this may be a sign of bleeding. Check the dressing status and consult with medical staff if necessary. | If there is bruising around the catheter, there is a possibility of blood clot formation. Contact 1599-3114 for medical advice. | Bruising around the catheter may be caused by minor bleeding during the insertion process. The bruise will fade over time, so there is no need for major concern. However, if the bruised area becomes swollen, red, or painful, this could be a sign of infection, and you should contact the hospital at 1599-3114. |
| **Q23** | Can I change the heparin flushing day? | Changing the heparin flushing schedule is not recommended. Flushing on the scheduled day helps prevent catheter occlusion. If you must change, try to get the flush as soon as possible. | Since you are currently receiving chemotherapy through a home pump, there is no need to flush heparin solution. Therefore, there is no need to change the schedule. | It's important to flush heparin on the scheduled day. Do you need to adjust your schedule? [If yes:] Maintaining a weekly interval is best, but if needed, it is safer to move it earlier rather than later. |
| **Q24** | I have chills or a high fever after heparin flushing. What should I do? | If you experience chills or high fever after heparin flushing, this may be a sign of infection. Consult with medical staff immediately to identify the cause and take appropriate measures. | If you experience chills or high fever after heparin flushing, there may be a risk of infection. If the fever is 38°C or higher, contact 1599-3114 for medical advice. | Is your fever 38°C or higher? [If no:] Can you recheck your temperature in one hour? If it rises above 38°C, you should visit the hospital within 24 hours. |
| **Q25** | I cannot flush the line because it feels blocked. What should I do? | If heparin flushing is difficult, the catheter may be blocked. Do not force the flush; consult with medical staff for appropriate measures. | If the catheter is blocked or heparin flushing is difficult, contact 1599-3114 for medical advice. | Do not force the flush. It would be best to visit the hospital to check whether the catheter is blocked. When can you come in? [Response:] In a few days. Can you come during weekday hours, 9 AM to 5 PM? You can visit the Hematology-Oncology outpatient clinic for assistance. |
| **Q26** | Should I dilute the heparin solution before use? | Heparin solution must be injected without dilution using a 10mL syringe. Diluting it may cause catheter occlusion. | Heparin solution should be used as-is without dilution. Diluting it may reduce the anticoagulant effect. | The heparin solution prescribed by our hospital is already diluted, so there is no need to dilute it further. Did you happen to dilute it before use? [If yes:] There is a risk of catheter occlusion. Please visit the hospital within 24 hours to have the heparin solution re-flushed. |
| **Q27** | I am using a chemotherapy infusion pump. Do I still need to flush with heparin? | If you are using a chemotherapy home pump, there is no need to flush heparin solution. The pump automatically administers heparin, so no additional flushing is required. | If you are using a chemotherapy home pump, there is no need to flush heparin solution. You can skip the heparin flush. | Since the home pump is already delivering chemotherapy, there is no need to flush heparin solution. |
| **Q28** | Should heparin be stored at room temperature? | Heparin solution should be stored at room temperature, away from direct sunlight. Do not store it in the refrigerator. | Heparin solution should be stored at room temperature. There is no need to refrigerate it. | Store it at room temperature. Did you happen to put it in the refrigerator? [If in the freezer:] Heparin stored in the freezer cannot be used. Please dispose of it. |
| **Q29** | I am running out of heparin. What should I do? | If your heparin supply is insufficient, visit a nearby pharmacy or hospital to purchase the required amount. It is best to prepare in advance to avoid delays in dressing changes. | If your heparin supply is insufficient, you should contact the hospital to purchase additional supplies. | You're running low on heparin. Have you checked with your care hospital whether they can prescribe more? If not, please contact our hospital. |
| **Q30** | What is the correct volume of heparin to flush? | The heparin flush volume for a PICC line depends on the catheter length. Typically, 5mL or 10mL is flushed. Confirm the exact volume with your medical staff. | The heparin flush volume depends on the catheter type. Typically 5cc is flushed, but the exact volume should be confirmed with your medical staff. | The heparin flush volume depends on the catheter length. For short catheters (5cm or less), flush 3cc; for longer catheters (over 5cm), flush 5cc. |
| **Q31** | Do I need to flush with heparin after chemotherapy infusion? | After chemotherapy injection, heparin solution should be flushed to prevent catheter occlusion. Always use a 10mL syringe for flushing. | After chemotherapy injection, there is no need to flush heparin solution. You can skip the heparin flush after chemotherapy. | It's important to flush heparin at the scheduled time. Do you need to adjust your schedule? [If no:] Continue with the original plan. |
| **Q32** | Can I take a half-body bath, or go to the swimming pool, public bath, sauna, or spa? | Half-baths, swimming pools, bathhouses, saunas, and spas increase the risk of infection at the insertion site and should be avoided. Shower quickly and keep the dressing site dry. | Half-baths, swimming pools, bathhouses, saunas, and spas increase the risk of infection at the insertion site and should be avoided. Shower quickly and always keep the dressing site dry. | Half-baths, swimming pools, bathhouses, saunas, and spas are not recommended. These places increase the risk of infection at the insertion site due to moisture. Full-body bathing should only be done two days after catheter removal, when there are no abnormalities. |
| **Q33** | Can I move my arm freely, exercise, or do daily work? | You can move your arm and exercise, but avoid putting excessive force on the catheter arm or lifting heavy objects. Normal daily activities are fine—just be careful not to overdo it. | You can move your arm, exercise, and work, but avoid lifting heavy objects or putting excessive force. Be careful not to let the catheter dislodge. | If you feel well, normal daily activities are fine. Do you lift objects heavier than 4 kilograms or carry heavy backpacks? [If no:] Then it's fine. |
| **Q34** | I sweated a lot after exercising, and the insertion site is damp. Do I need to change the dressing again? | If the insertion site area got wet with sweat after exercising, you must re-disinfect. After disinfection, protect the area with transparent film. | If the insertion site area got wet with sweat after exercising, you must re-disinfect. Can you safely proceed with the disinfection in a dry location? | Did the area where the dressing material is attached also get sweaty? [If yes:] Sweating can increase the risk of infection, so disinfection is needed. Please keep the disinfected area dry at all times. |
| **Q35** | Can I drive after catheter insertion? | Driving is possible. However, avoid driving if you are taking painkillers or medications that cause drowsiness. Also, be careful not to strain the catheter arm. | Driving is possible, but you should wait 24 hours after catheter insertion. Also, avoid driving if you are taking painkillers. | Yes, it's fine. Are you taking any narcotic painkillers? [If no:] You can drive with the catheter inserted. However, if you start taking narcotic painkillers, they may cause drowsiness, so you should avoid driving in that case. |
| **Q36** | Can I travel while having the PICC line? | Traveling is possible. However, prepare dressing supplies in advance and plan to maintain your dressing schedule. Be careful not to disrupt catheter care. | Traveling is possible, but it is important to bring sufficient dressing supplies and maintain the dressing schedule. For long trips, be careful to prevent catheter occlusion. | Yes, you can travel with the catheter. However, it is important to research hospitals where dressing can be done in advance and bring dressing supplies with you. |
| **Q37** | Can I take a flight while having the PICC line? | You can fly. However, since dressing supplies cannot be used in the cabin, prepare in advance and maintain your dressing schedule. | You can fly, but be careful to prevent catheter occlusion. Stay well hydrated during the flight and keep the insertion site dry with thorough disinfection. | Yes, you can fly. However, you should notify airport security about the catheter before going through screening. Do you have any other questions? |
| **Q38** | Can I wear an arm sleeve (compression sleeve) in daily life? | Wearing an arm sleeve/cover is possible. Just be careful not to wear it too tightly around the catheter arm. | You can wear an arm sleeve, but be careful not to make it too tight. Keep your arm comfortable at all times to prevent catheter occlusion. | Yes, you can wear an arm sleeve/cover in daily life. Wearing a sleeve or protective cover prevents the catheter from dangling and keeps it hidden, which may be more convenient. |
| **Q39** | Can I take a shower with the PICC line? | Showering is possible. However, use waterproof film to prevent the insertion site from getting wet, and re-disinfect after showering. | Showering is possible, but be careful not to let water touch the dressing site. Proceed with disinfection in a dry location before showering. | Yes, a light shower is possible. However, be careful not to let water get into the insertion site. You can use waterproof film and tissue, or a waterproof shower cover. Half-baths, swimming pools, bathhouses, saunas, and spas should be avoided due to increased moisture and infection risk. |
| **Q40** | Can I use walking aids such as a cane, stick, or crutches? | Canes, walking sticks, crutches, and other assistive devices can be used. Just be careful not to strain the catheter arm. | Yes, canes, walking sticks, and crutches can be used. However, avoid lifting very heavy objects or putting excessive force. | You can use a cane, walking stick, or crutches. However, be careful not to overuse the arm where the catheter is inserted. Avoid lifting heavy objects or activities that require extensive arm movement. |
| **Q41** | Can I sleep on the side where the catheter is inserted? | It is fine to lie on the side where the catheter is inserted. Just be careful not to put too much pressure on it. Maintain a comfortable position. | Yes, it is fine to lie on the side where the catheter is. Just be careful not to let the catheter dislodge. | It is fine to lie on the side where the catheter is. However, please secure the catheter well with adhesive tape to prevent dislodgement. |
| **Q42** | Can I receive manual therapy or massage with the PICC line? | Manual therapy or massage is possible. However, consult with a specialist to ensure the catheter arm is not strained. | Manual therapy or massage is possible, but it is best to avoid the side where the catheter is inserted. Be careful not to let the catheter dislodge. | If you receive manual therapy or massage, it is best to avoid the arm where the catheter is inserted. Please be careful not to use the catheter arm during the treatment. |
| **Q43** | When should I go to the emergency room in urgent situations? | Please go to the emergency room immediately in the following situations: a high fever of 38°C or above; severe pain at the catheter insertion site; the catheter coming out or being torn; severe bleeding; or difficulty breathing or decreased consciousness. For any other abnormal symptoms, please consult with your medical staff. | The situations that require an emergency room visit are as follows: (1) a high fever of 38°C or above; (2) severe bleeding; (3) difficulty breathing; (4) decreased consciousness; and (5) severe chest pain. If any of these symptoms occur, go to the emergency room immediately. Do you have any other questions? | The urgent situations that require an emergency room visit are as follows. If the catheter has come out or is at risk of coming out: if the catheter has completely come out, or if the insertion site has opened up severely so that the catheter is at risk of dislodging, you should visit the emergency room immediately. If the catheter is blocked and infusion is difficult: if the catheter is blocked when injecting heparin solution or medication and infusion is difficult, visit the emergency room if the problem persists even after taking measures to keep the catheter from being occluded. If there is severe pain or fever at the insertion site: if the insertion site is severely painful or shows signs of infection such as discharge of pus, consult your medical staff immediately and visit the emergency room if necessary. Systemic abnormal symptoms: you should also visit the emergency room if systemic abnormal symptoms appear, such as high fever (38°C or above), chills, severe headache, difficulty breathing, or decreased consciousness. When such situations arise, it is important to respond quickly to protect your health. |

**Supplementary Table S1.** **Pairwise exact McNemar tests for forced-choice evaluator preferences across the three models.**

| **Pairwise Comparison** | **Discordant Pairs** | **Exact McNemar *p*-value** | **Bonferroni-adjusted  *p*-value** |
| --- | --- | --- | --- |
| Model 1 vs Model 2 | (4, 2) | 0.687 | 0.999 |
| Model 1 vs Model 3 | (4, 34) | < 0.001 | < 0.001 |
| Model 2 vs Model 3 | (2, 34) | < 0.001 | < 0.001 |

**Supplementary Table S2. Quantitative performance comparison across eight evaluation criteria.**

| Evaluation Criteria | Model 1  (Fine-tuning) | Model 2  (Fine-tuning + RAG) | Model 3  (Fine-tuning + RAG + FSM) |
| --- | --- | --- | --- |
|  | Mean (SD) | Mean (SD) | Mean (SD) |
| Accuracy | 2.67 ± 0.58 | 1.67 ± 0.58 | 4.00 ± 1.00 |
| Clarity | 3.33 ± 0.58 | 2.33 ± 1.53 | 3.67 ± 0.58 |
| Actionability | 2.33 ± 0.58 | 2.33 ± 0.58 | 4.67 ± 0.58 |
| Completeness | 2.33 ± 0.58 | 1.67 ± 0.58 | 4.33 ± 0.58 |
| Efficiency | 4.00 ± 0.00 | 2.67 ± 1.53 | 3.33 ± 0.58 |
| Adaptability | 1.67 ± 0.58 | 2.33 ± 1.15 | 4.67 ± 0.58 |
| Safety | 2.00 ± 1.00 | 2.00 ± 1.00 | 4.33 ± 0.58 |
| Overall Quality | 2.67 ± 0.58 | 1.67 ± 0.58 | 4.00 ± 1.00 |

Abbreviation: SD, Standard Deviation
